# CNT-PUFs: Highly Robust and Heat-Tolerant Carbon-Nanotube-Based Physical Unclonable Functions †

**DOI:** 10.3390/nano13222930

**Published:** 2023-11-11

**Authors:** Florian Frank, Simon Böttger, Nico Mexis, Nikolaos Athanasios Anagnostopoulos, Ali Mohamed, Martin Hartmann, Harald Kuhn, Christian Helke, Tolga Arul, Stefan Katzenbeisser, Sascha Hermann

**Affiliations:** 1 Faculty of Computer Science and Mathematics, University of Passau, Innstraße 43, 94032 Passau, Germany; florian.frank@uni-passau.de (F.F.); nico.mexis@uni-passau.de (N.M.); nikolaos.anagnostopoulos@uni-passau.de (N.A.A.); tolga.arul@uni-passau.de (T.A.); 2 Center for Microtechnologies, Chemnitz University of Technology, Reichenhainer Str. 70, 09126 Chemnitz, Germany; simon.boettger@zfm.tu-chemnitz.de (S.B.); ali.mohamed@zfm.tu-chemnitz.de (A.M.); martin.hartmann@zfm.tu-chemnitz.de (M.H.); harald.kuhn@zfm.tu-chemnitz.de (H.K.); christian.helke@enas.fraunhofer.de (C.H.); 3 Center for Materials, Architectures and Integration of Nanomembranes (MAIN), Chemnitz University of Technology, 09107 Chemnitz, Germany; 4 Fraunhofer Institute for Electronic Nano Systems (ENAS), Technologie-Campus 3, 09126 Chemnitz, Germany; 5 Computer Science Department, Technical University of Darmstadt, Hochschulstraße 10, 64289 Darmstadt, Germany; 6 Center for Advancing Electronics Dresden (CFAED), 01062 Dresden, Germany

**Keywords:** Carbon NanoTube (CNT), Physical Unclonable Function (PUF), Nanomaterials (NMs), hardware security, security, privacy, Internet of Things (IoT)

## Abstract

In this work, we explored a highly robust and unique Physical Unclonable Function (PUF) based on the stochastic assembly of single-walled Carbon NanoTubes (CNTs) integrated within a wafer-level technology. Our work demonstrated that the proposed CNT-based PUFs are exceptionally robust with an average fractional intra-device Hamming distance well below 0.01 both at room temperature and under varying temperatures in the range from 23 ${}^{\circ }C$ to 120 ${}^{\circ }C$. We attributed the excellent heat tolerance to comparatively low activation energies of less than 40 meV extracted from an Arrhenius plot. As the number of unstable bits in the examined implementation is extremely low, our devices allow for a lightweight and simple error correction, just by selecting stable cells, thereby diminishing the need for complex error correction. Through a significant number of tests, we demonstrated the capability of novel nanomaterial devices to serve as highly efficient hardware security primitives.

## 1. Introduction

Physical Unclonable Functions (PUFs) have been proposed as low-cost security anchors in embedded systems [1], as they offer high uniqueness and unpredictable keys. PUFs have been successfully applied to the problems of device identification [2,3,4], key storage [5,6,7], and software protection [8,9]. A majority of PUF constructions currently utilise phenomena observed in silicon-based circuits, as these constitute the dominant architecture in the current electronics.

However, emerging electronic system architectures follow increasingly 3D integration concepts driven by further miniaturisation, increased performance, decreased energy consumption, and the implementation of further functionalities. In this context, nanodevices based on nanomaterials utilising the Complementary Metal–Oxide–Semiconductor (CMOS) technology find increasing attention, reflecting trends in semiconductor development roadmaps. In particular, multifunctional materials are preferred because they can take over multiple tasks in such systems, allowing for cost-efficient and parallel integration.

Promising nanomaterials to fulfil these requirements are single-walled Carbon NanoTubes (CNTs)—hollow cylinders consisting of sp^2^ hybridised carbon atoms in a hexagonal arrangement. Depending on the orientation between their tube axis and the underlying crystal lattice, they are either electrically conducting (metallic) or semiconducting. CNTs have already proven their capabilities for post-silicon electronics [10], as well as for gas, chemical, or bio-sensors [11], enabled due to their high intrinsic charge carrier mobility [12], high current carrying capacity, and mono-atomic surface [13]. At the same time, multi-walled CNTs can be utilised in hydrogels and aerogels in order to provide electromagnetic interference shielding [14,15,16]. In general, major achievements in technology integration have led to the ability to manufacture CNTs in scalable and CMOS-compatible procedures. This advancement has, in turn, resulted in major breakthroughs in the development of key electronic components, such as energy-efficient sub-10 nm Field-Effect Transistors (FETs) [17], record-speed nanotube-based random-access memory storage devices [18], and analogue high-performance radio frequency transistors [10]. In such a FET configuration, CNTs can even take over security tasks, which are highly demanded for the described heterogeneous system architectures. The interconnection of these novel devices and the transfer and storage of security-sensitive data necessitate strong cryptographic measures relying on robust and secure keys, like hardware-security anchors derived from Physical Unclonable Functions (PUFs).

**Figure 1:**
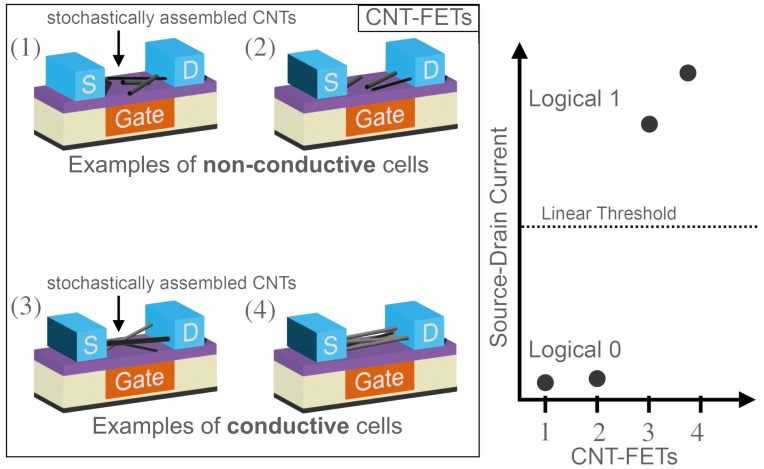
Conducting cells contain CNTs that bridge the source and the drain electrodes, resulting in a high source–drain conductance when applying a high negative or positive gate voltage such as ±2.5 V. In contrast, non-conducting cells exhibit very low source–drain conductance, independent of the applied gate voltage. This figure was adapted and modified from our previous work [19], which this work extends.

Therefore, we propose a binary PUF construction based on stochastically assembled CNTs produced by the printing processes as depicted in Figure 1. In combination with the subsequently deposited regular arrays of source (*S*) and drain (*D*) electrode pairs, separated by a small electrode gap, a certain proportion of those will be electrically connected by at least one CNT.

The conducting cells in Figure 1, specifically Cells (3) and (4), are treated as logical 1, whereas the non-conductive cells (Cells (1) and (2)) are treated as logical 0. Since those distinct states are clearly distinguishable in terms of electrical conductance, the PUF architecture is expected to be highly robust in contrast to many previously reported silicon PUFs, where errors have to be compensated by computationally intensive error-correction algorithms. Based on our construction, it is also expected that a high degree of robustness can be maintained even under varying environmental conditions, such as varying the ambient temperature. At the same time, several well-established PUF types suffer from insufficient temperature robustness, for example Latch or D-FlipFlop PUFs [20], along with DRAM retention [21] or ring-oscillator PUFs [22], limiting their field of application. Furthermore, in contrast to many other CNT-based PUFs, a passivation layer serves as an additional protection barrier, allowing for deployment in harsh environments. Consequently, this allows a robust key generation, for example in the automotive or industrial sector. Furthermore, a PUF implementation based on CNTs opens the door for various types of further hybrid nano-electronic devices, with additional cost-efficient intrinsic and highly robust CNT-based security anchors.

### 1.1. Contributions

This work focuses on the fabrication, read-out, analysis, and post-processing of data derived from Carbon NanoTube-based Physical Unclonable Functions (CNT-PUFs) utilising a new technological platform that was successfully validated for CMOS compatibility [23]. These CNT-PUFs were constructed from monolithic arrays of 144 Carbon NanoTube Field-Effect Transistors (CNT-FETs), which we will refer to as PUF cells in the remainder of this work. Depending on how CNTs are deposited on the surface of the underlying FET circuit, the respective circuit is conducting (signifying a logical 1) or non-conducting (signifying a logical 0). This PUF construction was already presented in our previous work [19], which this work extends. Based on the previous work, this article presents several additional innovative contributions:

- We conducted an additional temperature analysis of our CNT-PUFs, extending the application area of the CNT-PUFs to include even harsh environments. The investigated CNT-PUFs demonstrated very high stability with at most 2% unstable cells in the temperature range from $23\;{}^{\circ }C$ to $120\;{}^{\circ }C$.
- We performed a more-extensive security analysis of the raw CNT-PUF measurements. An analysis of the unpredictability of the raw PUF responses through an evaluation of the measurements by the statistical randomness tests of the National Institute of Standards and Technology (NIST) SP 800-22 test suite [24] was conducted. Our PUFs passed all applicable statistical randomness tests of the NIST SP 800-22 test suite [24], confirming the ability to use our CNT-PUFs as strong cryptographic primitives within cryptographic protocols.
- Finally, we present a very lightweight and efficient post-processing method that ranks all cells according to their stability and selects the most-stable cells for further use. By using this method, our CNT-PUFs are able to produce fully stable PUF responses.

### 1.2. Paper Organisation

In Section 2, we give an overview of previous publications in the field of CNT-based PUFs and discuss their relevance to the presented work. Section 3 provides relevant background information regarding the metrics utilised to assess the quality of the produced PUFs. Subsequently, Section 4 describes the CNT-PUF fabrication process and the relevant characterisation methodology in detail. In Section 5, we assess the quality of the implemented CNT-PUF at room and elevated temperatures using the metrics discussed in Section 3. In Section 6, a lightweight post-processing method to achieve completely stable PUF responses is proposed, and a brief outlook on the potential of our CNT-PUFs for future security applications is provided. Finally, Section 7 summarises the findings of this work.

## 2. Related Work

In this section, we provide a brief overview of some of the most-important works on carbon-nanotube-based PUFs and comment on their relevance to our work.

In 2014, Konigsmark et al. [25] examined the ability of two series of CNT-FETs to be used for the creation of PUFs through the comparison of their output currents. This work provided only simulation results for the temperature range between $-20\;{}^{\circ }C$ and $80\;{}^{\circ }C$ and reported an average intra-device Hamming distance of 1.9%, which is similar to the average Hamming distance value that our work achieved for different temperatures.

In 2016, Hu et al. [26] investigated the ability of self-assembled CNTs arranged in a 64×40 crossbar structure to serve as either a binary or a ternary PUF. For both cases, a mean intra-device Hamming distance of ≈3% was reported, which is higher than the 2% value reported in our work, which concerns temperature variations between $23\;{}^{\circ }C$ and $120\;{}^{\circ }C$. Additionally, the work of Hu et al. only examined the stability of the relevant CNTs through measurements at $25\;{}^{\circ }C$ and at $85\;{}^{\circ }C$.

In 2017, Moradi et al. [27] proposed novel CNT-based PUF types utilising either the voltage or the current output of CNT-FETs. This work provided simulation results for the temperature range between $0\;{}^{\circ }C$ and $100\;{}^{\circ }C$, demonstrating reliability values that would correspond to intra-device Hamming distances of at least 3.33%. In comparison, our work can achieve an intra-device Hamming distance of at most 2% in a temperature range of up to 120 ${}^{\circ }C$.

In 2018, Liu et al. [28] discussed the combination of a CNT-FET crossbar structure with the Lorenz chaotic system in order to provide PUFs that would be resistant to machine learning attacks. Again, only simulation results were provided. Furthermore, this work focused on the uniqueness, randomness, and unpredictability of the PUF responses, with no results being reported regarding the stability of these responses.

Also in 2018, Kumar et al. [29] proposed the use of multi-gate CNT-FETs for the creation of PUFs, in order to increase the number of bits that each CNT-FET could produce and, in this way, enhance the entropy of the overall PUF response, potentially resulting not only in ternary responses, but also up to base-17 PUF responses. However, this work focused on the uniqueness and the entropy of the produced responses and did not provide any results relevant to their robustness. Moreover, while individual CNT-FETs seem to have been fabricated for the purposes of this work, the relevant CNT-FET networks seem to have been only simulated.

A work by Moon et al. regarding PUFs produced by all-printed CNT networks that was published in 2019 [30] demonstrated no significant changes (only an overall 1.0% difference) regarding the reliability of the fabricated CNTs over 10,000 measurement cycles and 14 days. However, the relevant resistance characteristic of individual CNTs was reported to have changed up to 16.7%, and the results for temperature variations between $25\;{}^{\circ }C$ and $80\;{}^{\circ }C$ exhibited an average difference of up to 30%. In comparison, our work reports a very low number of unstable CNT cells for temperature variations between $23\;{}^{\circ }C$ and $120\;{}^{\circ }C$, around 2% of the overall structure examined. Additionally, the thin-film PUF architecture of the PUF proposed in [30] requires a much higher area per bit cell compared to our CNT-PUF.

Also, in 2019, Burzurí et al. [31] examined the ability of single-walled carbon nanotubes to be used for the creation of PUFs. This work demonstrated good results regarding the uniqueness and robustness of the fabricated PUFs, reporting intra-device Hamming distances of 6.3% after two weeks and 8.3% after two months. In comparison, our work allows for an average intra-device Hamming distance value of 0.1% at normal temperature (and 2% at differing temperatures) even after an extended time period.

In 2021, Srinivasu and Chattopadhyay [32] proposed a ternary-cycle-operator-based PUF utilising CNT-FETs. However, this work provided simulation results only regarding the uniformity of the relevant PUF responses and did not examine their robustness.

Most recently, in 2022, Zhong et al. [33] described the fabrication of identical pairs of ternary PUFs based on transferred Chemical-Vapour-Deposition (CVD)-grown CNTs on dual-line structures, which they referred to as “twin PUFs”. The individual PUFs were examined at room temperature and at $100\;{}^{\circ }C$ and exhibited high uniformity and uniqueness, as well as high consistency to their pairs, over a six-month period. The authors noted an intra-device Hamming distance value close to zero over six months, which appears to be an ideal result. However, we need to note that this result concerned a PUF whose characteristics are reproducible and, thus, predetermined during the manufacturing process (other examples of such PUFs include the ones described in [34] and in [35] and are also individually extremely robust), and not a PUF whose characteristics are not predetermined, but are only based on the slight intrinsic variations of the manufacturing process, leading to highly unpredictable responses, like the CNT-PUF that our work examined. Moreover, the intra-device Hamming distance was based on a comparably weak database of only two measurements. Ageing or fatigue arising from electrical loading upon cyclic readout was not considered. Considering parallel fabrication on large substrates such a CNT transfer process provokes biased and, thus, predictable PUFs. This is because CVD-grown horizontal CNTs lead to highly aligned unidirectional assemblies up to several millimetres along the CNT axis and, thus, cause repeating bit sequences in neighbouring PUFs.

We generally note an increased interest in CNT-based PUFs as demonstrated by the relevant literature. At the same time, however, we also observed that the relevant literature either fails to report on results regarding the stability of the examined CNT-based PUFs or the relevant average intra-device Hamming distance values are significantly higher than the 0.06% value that our work allows for, under normal conditions. Moreover, most of these works were based on simulation results, and the few of them that dealt with fabricated structures required either additional lithography steps for the definition of CNT deposition sites, e.g., [26], or sophisticated transfer processes, e.g., [33]. Furthermore, we noticed that the CNT transfer processes as described by Zhong et al. were hardly 200 mm wafer scalable, and the dielectrophoretic CNT integration from Burzurí et al. requires an additional expensive lithography and etching step. In contrast, our work presents a large-scale fabrication of 144-bit CNT-PUFs by highly scalable and automated printing-like processes on 200 mm wafers for stochastic CNT assemblies with a minimal amount of dispersion (<200 μL/wafer), featuring high uniformity and uniqueness. In addition, for the first time, the robustness of the CNT-PUFs was characterised in detail through repeated measurements utilising a cyclic thermal load between $23\;{}^{\circ }C$ and $120\;{}^{\circ }C$. Moreover, to the best of our knowledge, this work is the first to examine passivated CNT-PUFs. Passivation of p-type CNT-FETs is typically connected to reduced maximum currents and a shift of the transfer curve [36] due to the absence of the electrostatic p-doping from physisorbed oxygen molecules [37,38,39].

Nevertheless, device passivation is a crucial technology step to allow CNT-PUFs to be integrated into Application-Specific Integrated Circuits (ASICs), as shown in an earlier work [23]. In the end, a lightweight method for selecting stable CNT cells is proposed so that fully stable PUF responses can be generated. These advancements pave the way for cost-efficient heterogeneous security anchors in embedded systems.

## 3. Preliminaries: PUF Metrics

To assess the quality of the examined PUFs, several metrics are available:

- **Uniformity** describes how the bits of an individual PUF response are distributed. It is favourable to achieve an equal distribution of zeros and ones. A bias towards one of the values leads to higher predictability of the responses, compromising the overall security of the resulting response. Uniformity is evaluated by the Hamming weight $HW(R_{i}^{x}\left(C\right))$, which measures the number of ones in a binary PUF response $R_{i}\left(C\right)$ of PUF instance *i* to challenge *C* that has been received at normalised time *x*. The normalised average ${\mathrm{HW}}_{i}$ for a PUF *i* is calculated as follows:
  (1) $${\mathrm{HW}}_{i}=\frac{1}{l}\;\cdot \;\sum_{x=1}^{l}\frac{HW(R_{i}^{x}\left(C\right))}{\left|PUF\right|}\;,$$
  where *l* is the number of responses collected for a challenge *C* at all normalised time values and |PUF| the length of the PUF responses of PUF *i*. The ideal value of 0.5=50% signifies, on average, a perfectly balanced distribution of zeros and ones within the PUF responses of PUF *i*.
- **Uniqueness** measures the independence of responses originating from different PUFs for the same challenge *C*. This property is typically evaluated by the average fractional inter-device Hamming distance ${\mathrm{HD}}_{inter}$, which specifies the normalised average number of bit positions that differ in the responses of all different PUF instances, with response $R_{i}^{x}\left(C\right)$ being the response of PUF *i* to challenge *C* at normalised time *x* and response $R_{j}^{y}\left(C\right)$ being the response of PUF *j* to challenge *C* at normalised time *y*. Essentially, the Hamming distance between $R_{i}^{x}\left(C\right)$ and $R_{j}^{y}\left(C\right)$, denoted by $HD(R_{i}^{x}\left(C\right),R_{j}^{y}\left(C\right))$, is equal to $sum(R_{i}^{x}\left(C\right)\;⊕\;R_{j}^{y}\left(C\right))$, counting the number of different bit positions when comparing the two binary vectors $R_{i}^{x}\left(C\right)$ and $R_{j}^{y}\left(C\right)$. For PUF responses of equal length |PUF| (if the PUF responses differ in their length, the length of the shorter response is chosen for their comparison, and the number of bits remaining from the longer response is added to the comparison result, to calculate the overall ${\mathrm{HD}}_{inter}$), with *k* being the number of devices measured, *m* the number of responses collected from device *i* for a response *C* at all normalised times, and *n* the number of responses collected from device *j* for the response *C* at all normalised times; ${\mathrm{HD}}_{inter}$ is given by:
  (2) $${\mathrm{HD}}_{inter}=\frac{2}{k\;\cdot \;(k-1)}\;\cdot \;\sum_{i=1}^{k-1}\sum_{j=i+1}^{k}\left(\frac{1}{m\;\cdot \;n}\;\cdot \;\sum_{x=1}^{m}\sum_{y=1}^{n}\frac{HD(R_{i}^{x}\left(C\right),R_{j}^{y}\left(C\right))}{\left|PUF\right|}\right)\;.$$
  A value of ${\mathrm{HD}}_{inter}=0.5=50\%$, meaning that, on average, half of the bits of the response of one PUF instance have a different value from the bits of all other instances, indicates the highest possible degree of uniqueness.
- The property of **robustness** describes the stability of PUF responses originating from the same PUF, for a given challenge *C*, under repeated PUF measurements. This property is evaluated using the average fractional intra-device Hamming distance ${\mathrm{HD}}_{intra}$, which measures the normalised average number of bit positions that differ between each two responses $R_{i}^{x}\left(C\right)$ and $R_{i}^{y}\left(C\right)$ stemming from the same device *i* produced using the same challenge *C* and captured at different normalised times *x* and *y*. ${\mathrm{HD}}_{intra}$ is given by:
  (3) $${\mathrm{HD}}_{intra,i}=\frac{2}{l\;\cdot \;(l-1)}\;\cdot \;\sum_{x=1}^{l-1}\sum_{y=x+1}^{l}\frac{HD(R_{i}^{x}\left(C\right),R_{i}^{y}\left(C\right))}{\left|PUF\right|}\;,$$
  where *l* is the number of responses collected for a challenge *C* at all normalised time values and |PUF| the length of the PUF responses of PUF *i*. A value of ${\mathrm{HD}}_{intra}=0$, meaning that all of the bits are identical in all responses, indicates the highest possible degree of stability and, hence, robustness.
- Another important property is the **unpredictability** of the generated responses. One pre-condition for high unpredictability is a uniform distribution of logical ones and zeros as explained previously. In addition, single bits of the PUF response should be free of correlations. Unpredictability is typically evaluated by a collection of statistical tests for randomness performed on the PUF responses. This paper used the well-known NIST SP 800-22 test suite [24]. This test suite consists of various statistical tests applied to presumably random strings, to check if they meet the entropy requirements to be used as primitives in cryptographic protocols.

## 4. Fabrication and Implementation of CNT-PUFs

### 4.1. Wafer-Level Fabrication Process

Our CNT-PUFs were fabricated under clean-room conditions utilising a nano-device platform technology for large FET arrays on 200 mm silicon wafers. This platform includes on-top CMOS-compatible surface micro-machining processes with standard projection lithography in line with scalable nanomaterial integration processes for the fabrication of more than 2000 CNT-PUFs per wafer in parallel and at an almost 100% yield. The fabricated sample configuration displays an intermediate processing step aiming for crossbar architectures. There, individual CNT-FETs will be interconnected by a 12×12 electrode matrix to form a 144-bit PUF.

**Figure 2:**
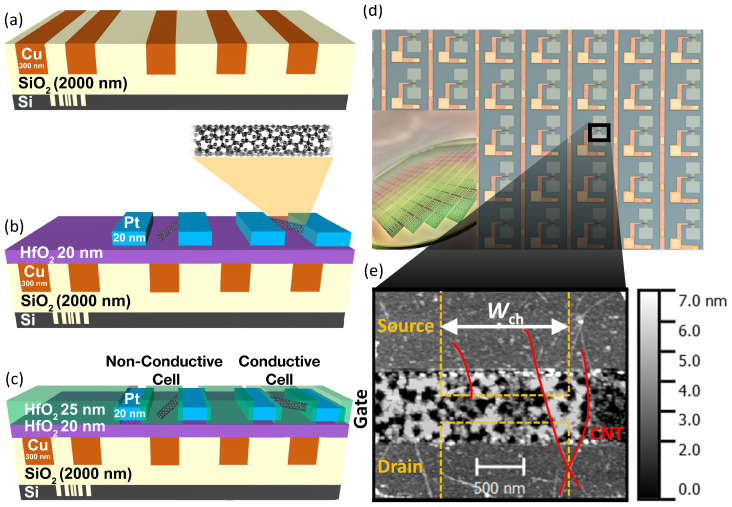
Schematic cross-section view of the fabrication process of passivated CNT-FET arrays (**a**–**c**), light microscope top-view image of a section of a CNT-PUF (**d**), and an AFM image of stochastically assembled CNTs (**e**). The inset in (**d**) shows a photograph of the finally processed 200 mm wafer. This figure was adapted and modified from our previous work [19], which this work extends.

Figure 2 shows the simplified processing scheme on a schematic cross-section of PUF cells (a–c), a top-view light microscope image of a CNT-PUF section together with a photograph of a fully processed 200 mm wafer (d), as well as an Atomic Force Microscopy (AFM) image showing stochastically assembled CNTs over a buried gate electrode (e).

First, buried electrodes for back gates and wirings were integrated using a copper single damascene technology on 2 μm thermally grown silicon oxide layers. There, silicon dry etching was used to etch the trenches. After this process, a metal layer stack was deposited consisting of 20 nm of titanium and 20 nm of titanium nitride as barrier and adhesion layers followed by 70 nm of copper as a seed layer for the subsequent electro-chemical deposition step of copper. Using chemical–mechanical polishing, the copper and the barrier were polished away, revealing 500 nm-wide gate electrodes with a dishing below 20 nm, as shown in Figure 2a.

Next, the high-k gate-dielectric was deposited by plasma-enhanced Atomic Layer Deposition (ALD) of 20 nm of hafnium dioxide. Afterwards, highly-enriched semiconducting CNTs with a mean CNT length of 2 μm and a diameter in between 1.2 nm and 1.4 nm (IsoSol-S100^®^ [40] from Nanointegris Technologies Inc., which is located in Boisbriand, QC, Canada) were integrated by an automated printing-like method. The density of the stochastically assembled CNTs was controlled by the CNT dispersion concentration and deposition process parameters such as the dispersion flow rate and the printing velocity. The CNT density was analysed by AFM and scanning electron microscopy. Detailed information on the CNT raw material, the CNT integration process, and the CNT-FET passivation is provided within the Supplementary Materials file.

The wafer was subsequently immersed into toluene with a 1 vol.% of tetrafluoridic acid, into acetone, and into toluene (at $50\;{}^{\circ }C$) for 30 min in order to remove residual polymers. The CNT assembly was then structured using lithography, inductively coupling oxygen plasma etching, and wet-resist removal in acetone with ultrasonic treatment. Afterwards, the contact electrodes were realised via Electron-Beam Lithography (EBL) with a subsequent ion-beam sputter deposition of 0.5 nm of chromium and 20 nm of platinum and an ultrasound-supported lift-off process in acetone.

A channel length of 500 nm was realised, and the contact electrodes were precisely aligned over the gate electrode, as shown in Figure 2b. The channel width $W_{\mathrm{ch}}$ was varied to be 500 nm, 1000 nm, and 2000 nm, respectively. Thus, the average number of CNTs per device and, consequently, the number of devices with an electrical connection between the electrodes can be controlled.

Afterwards, the wafer was annealed at $500\;{}^{\circ }C$ under an argon–hydrogen atmosphere at a pressure of 5 mbar for 30 min. To encapsulate the samples against ambient conditions and humidity, a thermal ALD of 25 nm of hafnium dioxide was conducted on the cells. The hafnium dioxide passivation over the source and drain contact pads and the gate dielectric over the gate contact pad were then etched by reactive ion etching, as visualised in Figure 2c.

### 4.2. CNT-PUF Characterisation

After CNT integration, the wafer was characterised by evaluating AFM topography images to determine the realised CNT density depending on the different integration process parameters. This way, the number of CNTs crossing arbitrary cross-sections drawn within the device channel in the parallel direction of the later-defined source and drain electrode edges was counted, and a CNT line density was extracted and used for the studies contained in Section 5.

**Figure 3:**
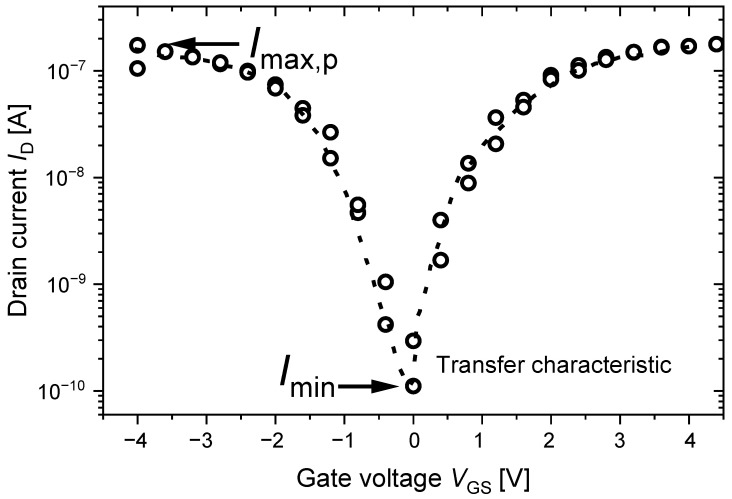
Illustration of a transfer curve applying pulsed sweeps across the gate voltage $V_{GS}$, between −4 V and 4 V.

After finishing the fabrication, each individual cell of the final CNT-PUF was electrically characterised using a Cascade PA200 semi-automated probe station and a Keithley 4200A-SCS semiconductor parameter analyser. These data provided the basis for the evaluation in Section 5. A representative transfer curve of a CNT-FET reflecting a conductive cell is shown in Figure 3. Such ambipolar transfer curves were captured in particular for the investigations in Section 5.3. There, pulsed transfer curves were measured in order to gain a complete understanding of the temperature dependence for the CNT-FETs. In this measurement procedure, a voltage pulse with $-V_{\mathrm{GS}}$ was applied before the actual measurement at $V_{\mathrm{GS}}$ to unload the charge traps and, thus, suppress hysteresis effects. The maximum current for the p-branch $I_{D,\max,p}$ was extracted from these curves. Extracting multiple transfer characteristic measurements requires a considerable time investment. Therefore, for all investigations, fast measurements using a single set of bias voltages were performed to ensure statistically profound results within an acceptable amount of time. There, the maximum drain current $I_{D,\max,p}$ was captured at $V_{GS}=-2.5V$ and $V_{DS}=-1V$. For the robustness measurements, we limited ourselves to 28 cycles. After this number of measurements, the PUF response remained essentially unchanged, so this was found to be the best compromise between time consumption and the number of repeated measurements.

### 4.3. CNT-PUF Quantisation

The fabricated CNT-PUFs provide a binary response, which is extracted through quantisation of analogue measurements of the PUF cells. Therefore, for each CNT-PUF, we assigned the measured values of each of the 144 PUF cells to one of two classes, representing logical 0s and logical 1s. Here, non-conducting cells are defined as logical 0 and conducting cells are defined as logical 1, such that a CNT-PUF returns a binary response $R_{p}$ with $|R_{p}|:=144$. This assignment was based on the quantisation of $I_{D,\max,p}$ gained from the measurements for each PUF cell using a threshold $I_{th}$.

The proposed quantisation procedure accepts an array of measurements $M_{p}:=\left\{I_{D,\max,p}^{0},I_{D,\max,p}^{1},\ldots ,I_{D,\max,p}^{143}\right\}$, where each element is the current $I_{D,\max,p}$ of each cell of a single CNT-PUF *p*, and compares each $I_{D,\max,p}$ with a threshold $I_{th}$. If $I_{D,\max,p}<I_{th}$, a logical 0 is added to the binary PUF response $R_{p}$; otherwise, a logical 1. Finally, a 144-bit binary PUF response is returned.

**Figure 4:**
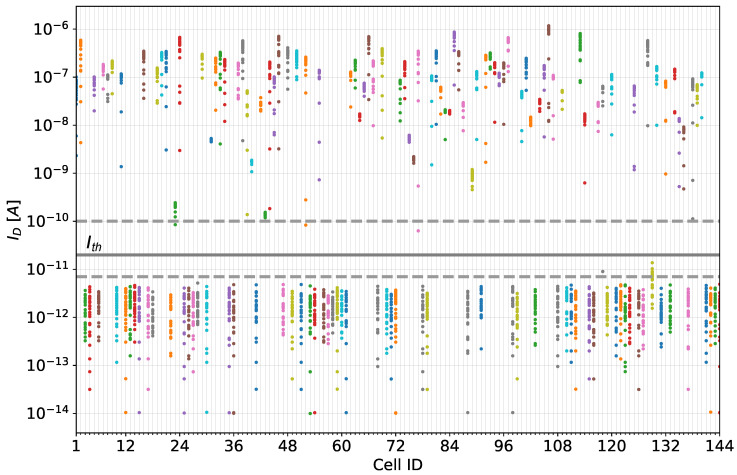
Distribution of 28 measurements of the source–drain current of each cell when applying $V_{GS}=-2.5\;V$ and $V_{DS}=-1\;V$. One can retrieve the stability of each cell from this figure, as well as the overall distribution of conducting and non-conducting cells of the examined device, based on a broad window within which the threshold $I_{th}$ can be set. The window is indicated by light grey lines and an exemplary threshold by a dark grey line. This figure was adapted from [41].

In order to reliably assign the measured values to a class under repeated measurements, a suitable value for the threshold $I_{th}$ must be determined. In Figure 4, the distribution of raw measurements of a single CNT-PUF (consisting of 144 PUF cells) is shown. The light grey dashed lines indicate the window of acceptable thresholds, where conducting and non-conducting cells can be clearly separated with only a few outliers. A threshold $I_{th}$ can be set within a broad range between $I_{D}=7\;pA$ and $I_{D}=100\;pA$. By placing the threshold $I_{th}$ within this window, a stable PUF response can be generated for almost all measurements and PUF cells, as indicated by the dark grey line denoting an exemplary threshold in Figure 4. The selection of the best $I_{th}$ value to achieve the most-stable responses is discussed in Section 5.2.4.

## 5. Evaluation of the Fabricated CNT-PUFs

### 5.1. CNT-PUF Design with Highly Distinguishable Responses

A basic requirement for a robust PUF is the generation of highly stable responses. Therefore, an important design goal of CNT-PUFs is to be able to reliably classify CNT-PUF cells into conductive and non-conductive cells among repeated measurements. To determine the best CNT-PUF design that yields the optimal distribution of responses, we studied cells with three different channel widths $W_{ch}$, namely 500 nm, 1000 nm, and 2000 nm. The channel’s position and width are shown in Figure 2e. The channel width, due to its geometry, has a decisive influence during the fabrication of the PUF on whether the corresponding cell becomes conductive or non-conductive, as the probability for a conductive cell increases with increasing width at a constant density of deposited CNTs.

**Figure 5:**
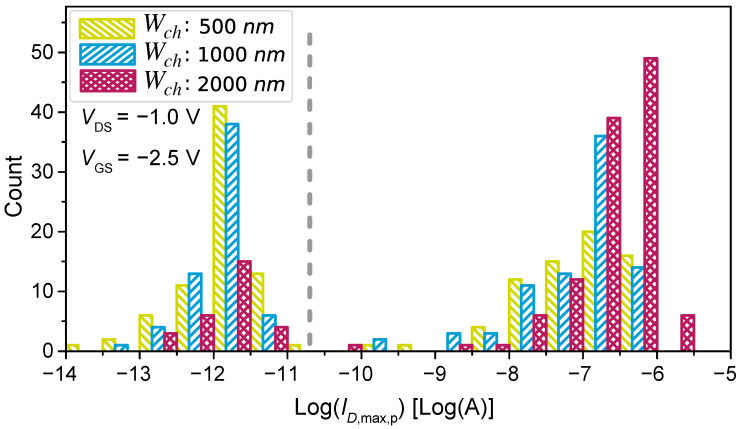
Distribution of the maximum drain current for 144-bit structures of different channel widths. This figure was adapted and modified from our previous work [19], which this work extends.

The electrical behaviour of cells having different channel widths is shown in Figure 5. The number of cells conducting a certain maximum drain current $I_{D,\max,p}$ is plotted on a logarithmic scale. It can be seen that, despite the different distribution of conducting and non-conducting cells for each channel width, there is a clear separation between these two classes independent of the channel width. The conducting cells are located in the nA to μA range ($10^{-9}A<I_{D,\max,p}<10^{-5}A$) and the non-conducting devices in the sub-nA range ($10^{-11}A$ and lower).

In between these two major groups of cells, we can also see a few cells with ambiguous drain current values. Since the maximum drain current of these cells is near the threshold voltage for quantisation, they can cause bit flips and, thus, instabilities in the PUF response. Section 6 discusses the treatment of these cells. Figure 5 also shows the decisive influence of the channel width on the ratio between conducting and non-conducting cells. The distribution of the maximum drain current for conducting devices ($I_{D,\max,p}>1.0\;nA$) shows a log-normal behaviour with an average of (−7.0±0.6)Log(A) for a channel width of 500 nm, (−7.0±0.7)Log(A) for a channel width of 1000 nm, and (−6.6±0.5)Log(A) for a channel width of 2000 nm. For a channel width of 2000 nm, a slight increase in the maximum observed $I_{D,\max,p}$ can be seen (Figure 5). This stems from the increased probability of parallel conductive paths occurring when multiple CNTs are deposited so that they bridge the CNT-FET channel and confirmed that their occurrence correlated with the channel width. The described phenomenon can be observed by looking at the two CNTs in Figure 2e, which form a bridge between the source and drain electrodes.

**Figure 6:**
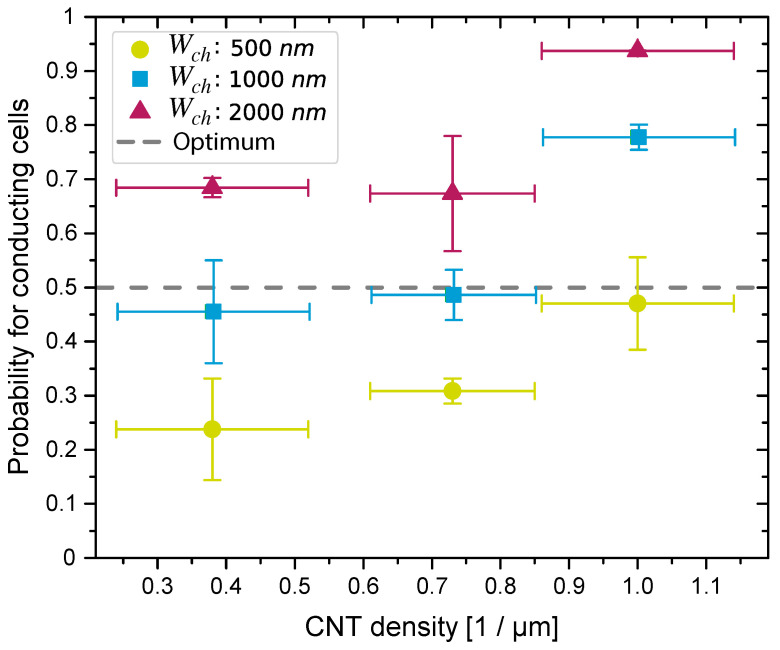
Probability of conducting cells depending on CNT density $\rho_{\mathrm{CNT}}$ and channel width $W_{\mathrm{ch}}$, for $V_{\mathrm{GS}}=-2.5\;V$ and $V_{\mathrm{DS}}=-1.0\;V$. This figure was adapted and modified from our previous work [19], which this work extends.

Another process parameter we investigated during the fabrication of the PUF cells was the relationship between the channel width, the CNT density, and the probability that the relevant CNT-FET is conductive. For this study, the threshold current was set at 20 pA. Figure 6 shows the probability for conductive cells depending on the CNT density and different channel widths. We see that, regardless of the channel width, as the density of the assembled CNTs increased, the frequency of conducting cells increased as well. Initially we assumed a CNT density $\rho_{\mathrm{CNT}}=0.5\;\cdot \;W_{\mathrm{ch}}^{-1}$ to target the highest binary uniformity for a given $W_{\mathrm{ch}}$. However, the behaviour was not as linear as expected. Obviously, the frequency of conducting cells for structures with a channel width of 2000 nm and a CNT density $\rho_{\mathrm{CNT}}=0.74\;\mu m$^−1^ is far below our expected value, whereas the probability of conducting cells for structures with a channel width of 500 nm fits well with the model we used. This deviation can possibly be explained by the loss of CNTs during wet-chemical cleaning, which potentially is more pronounced for CNT network assemblies. There, stacked CNTs exhibited lower Van der Waals interactions with the underlying substrate and, thus, cannot withstand the shear forces appearing during the rinsing and drying procedures. Other possible error sources include inhomogeneous CNT assemblies and an inappropriate line density model. The latter can lead to deviations because the channel length is 500 nm and, thus, falls within the range of the CNT length (1 μm).

Overall, the results presented in Figure 6 clearly show that the ratio of conducting cells can be controlled to a significant extent by the integration process and cell design. In particular, we can see that cells with a channel width of $W_{ch}=1000\;nm$ and a CNT density of $\rho_{\mathrm{CNT}}=0.74\;\mu m$^−1^ provided a nearly uniform distribution of conducting and non-conducting cells, which is, thus, almost ideal for the construction of binary PUFs.

### 5.2. Evaluation of CNT-PUFs under Normal Conditions

For the following evaluation, we applied the quality metrics for PUFs presented in Section 3 to the raw measured data of the selected CNT-PUFs. All measurements used in this section were obtained under a constant temperature of $23\;{}^{\circ }C$ in a laboratory.

#### 5.2.1. Uniformity

**Figure 7:**
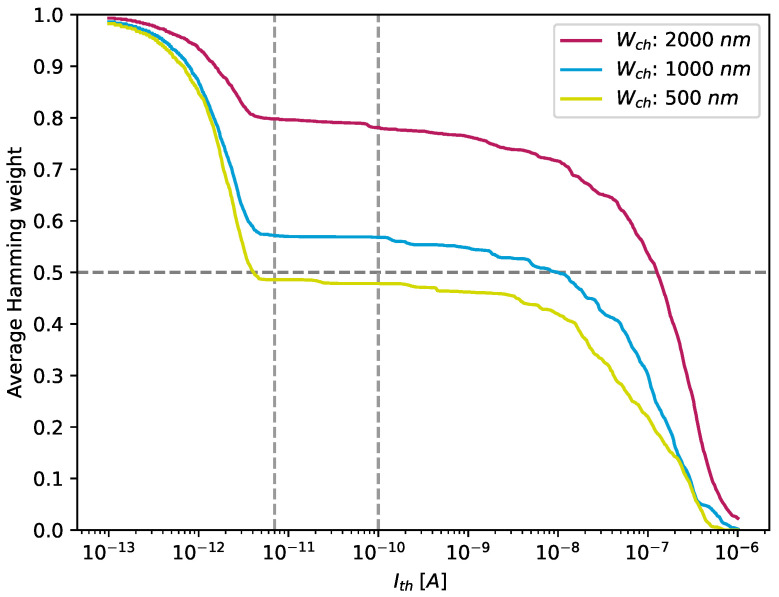
Hamming weight calculated for the PUF responses of three CNT-PUFs, one for each of the three different channel widths, depending on a threshold value of 0.1 pA to 1 μA. The grey lines indicate the range where the amount of both 0 s and 1 s remains almost constant and is the same as in Figure 4, giving the best stability.

We first checked whether ones and zeros were equally distributed in each PUF response. This property was evaluated by applying the quantisation algorithm introduced in Section 4.3 to the raw measurements of each individual CNT-PUF. Afterwards, the number of non-conducting cells (logical 0) and conducting cells (logical 1) was counted for each individual CNT-PUF, from which the Hamming weight was calculated. To optimally set the threshold $I_{th}$ so that a nearly uniform distribution of PUF bits can be achieved, this metric was evaluated for different thresholds $I_{th}$. Figure 7 illustrates the Hamming weight of three PUF responses with different channel widths, denoted as $W_{ch}$.

For all measurements, $V_{D}=-1\;V$ and $V_{GS}=-2.5\;V$ were applied to the circuit. As we can see, almost all CNT-PUFs, except the ones with $W_{ch}=2000\;nm$, achieved an almost uniform distribution of PUF bits when the threshold was set within the range between $I_{D,\max,p}=7\;pA$ and $I_{D,\max,p}=100\;pA$ (indicated by dashed vertical lines in the figure). By adjusting the threshold, more cells were classified as conductive cells when choosing a lower threshold, while higher thresholds led to more non-conductive cells and, thus, to a lower Hamming weight. As a consequence, Figure 7 exhibits a step-like shape. Moreover, we can see that a channel width of 2000 nm led to a higher amount of conducting cells and, thus, a bias towards logical 1s. The fact that the PUFs can maintain this almost ideal ratio of ones and zeros over a wide range of threshold values turned out to be a good indicator for robust PUF responses, as further indicated in Section 5.2.4.

**Figure 8:**
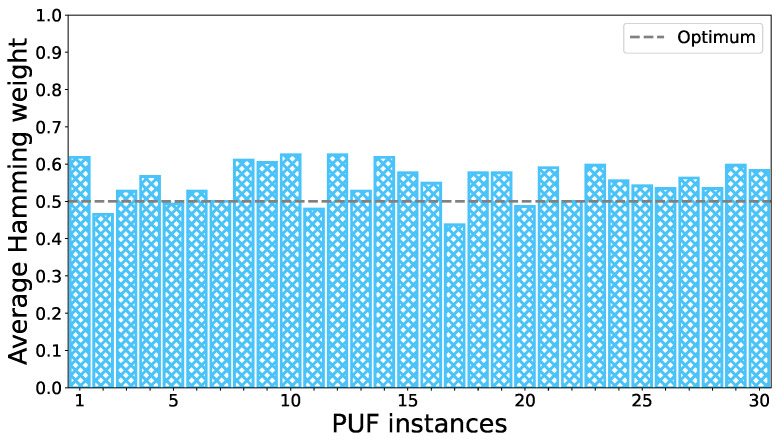
Histogram of the relative amount of conductive cells of 30 CNT-PUFs with a 1000 nm channel width. The classification of conductive and non-conductive cells is based on a threshold of $I_{th}=20\;pA$. This figure was adapted and modified from our previous work [19], which this work extends.

We can derive that, by using a threshold value of $I_{th}=20\;pA$, an almost uniform distribution of non-conducting (44.71%) and conducting cells (55.29%) for cells with $W_{ch}=1000\;nm$ can be achieved. These values are close to the optimal value of 50% for each class. In Figure 8, the ratio of non-conducting and conducting cells for each of the 30 measured CNT-PUFs is visualised for a threshold of $I_{th}=20\;pA$. It can be seen that no CNT-PUF exhibited an average fractional Hamming weight higher than 62.5% or lower than 43.75%.

#### 5.2.2. Uniqueness

**Figure 9:**
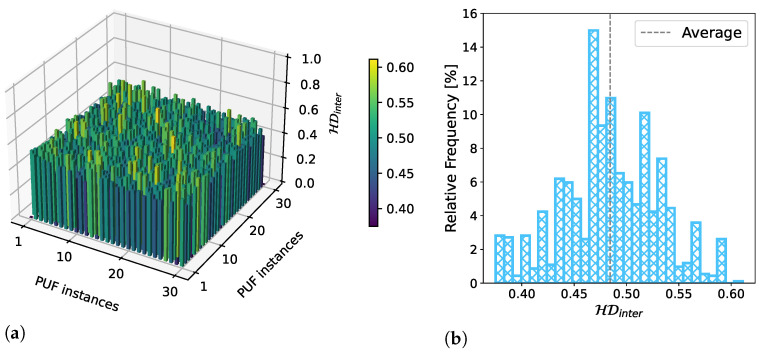
Figure (**a**) shows the comparisons of each of the 30 CNT-PUFs with each other using the inter-device Hamming distance. This figure was adapted and modified from our previous work [19], which this work extends. Figure (**b**) visualises the distribution of these inter-device Hamming distances as a histogram. Each bar represents the relative amount of comparisons within that area. The average inter-device Hamming distance is 0.48, which is close to the optimum.

To evaluate the uniqueness of our CNT-PUFs, the 144-bit binary responses returned from each of the 30 CNT-PUFs were compared. We used the inter-device Hamming distance introduced in Section 3 to perform this comparison. Figure 9a shows the Hamming distances of all 30 CNT-PUFs to each other (inter-device Hamming distances), excluding the Hamming distance of each PUF to itself (intra-device Hamming distances). The results showed that almost all values were centred on the optimal value of 0.5. As we can see from the box plot in Figure 9b, the exact average inter-device Hamming distance was 0.48, with no inter-device Hamming distance above 0.62 or below 0.37. These results demonstrated a high level of uniqueness.

#### 5.2.3. Unpredictability

In order to test whether the generated raw responses of our CNT-PUF were suitable for use as cryptographic keys, we checked their unpredictability. Unlike uniformity or uniqueness, no single formula can be used for checking this metric. Usually, a whole series of different statistical tests is performed to assess the unpredictability of PUF responses. For this purpose, we used the well-established NIST SP 800-22 test suite [24], applied on individual binary PUF responses. Table 1 indicates how many of the 30 PUF responses passed a specific test. The average *p*-value is provided for each test applied on all responses. According to the criteria defined in the NIST SP 800-22 test suite [24], a sequence is deemed random if its corresponding *p*-value ≥0.01. However, from these criteria, it can be seen that almost all PUF responses passed the most-important tests, indicating that they had a high degree of unpredictability and were, therefore, suitable for use as primitives in cryptographic applications. Nevertheless, it was not possible to obtain statistically sound values from the Random Excursions and Random Excursions Variant tests for our CNT-PUFs due to the high recommended input lengths above 144 bits, as shown in Table 1.

Furthermore, each PUF response can be represented as a two-dimensional array (matrix), facilitating the application of dedicated spatial correlation analysis techniques. Results obtained using established metrics such as Moran’s *I*, Geary’s *C*, Join Count $J_{BW}$, and even the Getis–Ord *G* were published in a paper by Mexis et al. [42]. The findings demonstrated a consistent positive spatial auto-correlation in four out of four PUF responses obtained from our proposed PUF structure. This auto-correlation primarily arises from the clustering of logical 1s.

#### 5.2.4. Robustness

**Figure 10:**
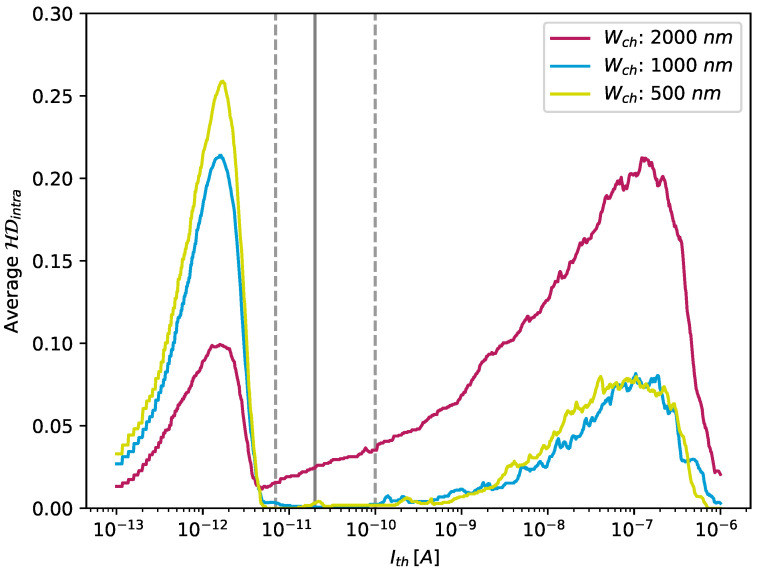
Amount of stable measurements calculated for different thresholds over the range of 0.1 pA to 1 μA, evaluated using 28 repeated measurements of three different 144-bit CNT-PUFs, one for each of the three different channel widths. The dashed grey lines indicate the range in which the value of ${\mathrm{HD}}_{\mathrm{intra}}$ is almost optimal and is the same as in Figure 4. This figure was adapted and modified from our previous work [19], which this work extends.

When prompted multiple times by an identical challenge *C*, the robustness of a PUF *p* guarantees that the response is always $R_{p}\left(C\right)$. To determine the robustness, we calculated the intra-device Hamming distance between PUF responses to the same challenge. As mentioned in the previous section, the cells of the CNT-PUFs were fabricated in such a way that conductive and non-conductive cells can be distinguished from each other exceptionally well. Figure 10 plots the relative proportion of unstable responses that occurred in 28 repeated measurements of three different 144-bit CNT-PUFs for all three channel widths as a function of the threshold value selected in each case.

It can be seen that a wide range of thresholds can be set in order to ensure stable responses: Values for $I_{th}$ between 7 pA and 100 pA led to almost no misclassified responses, while $I_{th}$ values outside this range led to unstable responses, as is evident in Figure 10. If the threshold was set in the former range, the maximum (worst) intra-device Hamming distance was 0.03=3%, which corresponds to a maximum of five erroneous bits. This result indicated that, even in the worst case, within the aforementioned range, fully stable responses of at least 139 bits can be achieved, which means that cryptographic tokens, such as keys, of a length of 128 bits can be easily derived from the examined CNT-PUFs. Overall, based on the analysis of the 28 repeated measurements, an average intra-device Hamming distance of 0.06% can be achieved with a threshold $I_{th}=20\;pA$. The number of measurement cycles represents a compromise aimed at capturing a statistically profound database to allow a suitable PUF evaluation within a reasonable measurement time. Moreover, considering the high ratio between conductive and non-conductive cells, as illustrated in Figure 4, it is unlikely that additional factors, such as noise introduced by additional measurements, have an impact on the classification result, assuming that the threshold $I_{th}$ is positioned within the window of high stability, indicated by the dashed grey lines in Figure 10.

### 5.3. Heat-Tolerant CNT-PUFs

Due to their preferred use as lightweight safety components in embedded systems, PUFs are often exposed to various demanding environmental conditions. Most commonly, these are conditions characterised by temperature fluctuations, typically in industrial or Internet-of-Things (IoT) applications. Regardless of how challenging these conditions may be, the PUFs should produce a stable response under all circumstances. To this end, in this work, we investigated the tolerance of CNT-PUFs to high heat, up to $120\;{}^{\circ }C$, well above the usual operating temperature limits of $70\;{}^{\circ }C$ for commercial semiconductor devices and $85\;{}^{\circ }C$ for industrial semiconductor devices and close to the operating temperature limit of $125\;{}^{\circ }C$ for military semiconductor devices [43]. Our CNT-PUF can be deployed in all of these domains, significantly exceeding the temperature robustness of consumer and industrial applications.

**Figure 11:**
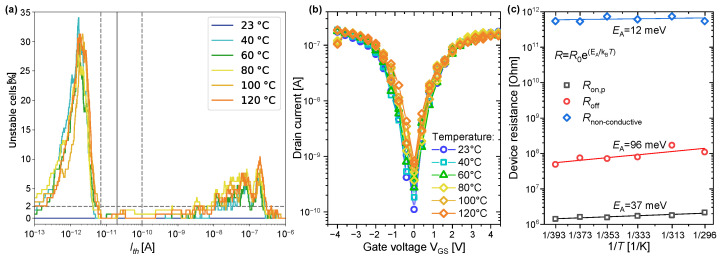
Figure (**a**) shows the number of unstable cells (in comparison to the $23\;{}^{\circ }C$ measurement) evaluated across the temperatures in *T*. The dashed grey lines indicate the range in which the value of unstable cells is almost optimal and is the same as in Figure 4. Figure (**b**) shows the deviation of the drain current $I_{D}$ under temperatures *T* when varying $V_{GS}$. In Figure (**c**), the extraction of activation energies for temperature-induced resistance changes underlines the excellent heat tolerance of the PUF construction. $R_{\mathrm{on},p}$ and $R_{\mathrm{off}}$ were extracted from a representative CNT-FET (conducting cell), whereas $R_{\mathrm{non}-\mathrm{conductive}}$ mirrors the average temperature dependence of 81 non-conductive cells.

In particular, we investigated the robustness of our CNT-PUFs at the temperatures $T:=\left\{23,40,60,80,100,120\right\}{}^{\circ }C$. Here, we always compared the PUF responses at a temperature of t∈T, with the reference measurement obtained at a temperature of $23\;{}^{\circ }C$. Figure 11a shows the fraction of unstable cells of a single 144-bit CNT-PUF instance captured at all temperatures t∈T as a function of the threshold $I_{th}$. As we can see, the temperature curves show no significant difference.

In addition, the wide window of usable thresholds obtained from Figure 10 can be confirmed for all temperature settings. This means that, even when comparing, within this window, responses at $23\;{}^{\circ }C$ to those at temperatures up to $120\;{}^{\circ }C$, at most 2% of cells were unstable, as shown in Figure 11a. We further investigated the temperature dependence of individual PUF cells. The maximal current $I_{D,\max,p}$, as well as the minimal current $I_{D,\min}$ for conductive cells exhibited an increase with increasing temperature, which is typical for semiconductors. This can be seen in the transfer curves of a representative CNT-FET in Figure 11b measured at different temperatures, as well as in the Arrhenius plot in Figure 11c. There, the resistance at the maximal p-current ($R_{\mathrm{on},p}$) and at the minimal current ($R_{\mathrm{off}}$) of the CNT-FET is logarithmically plotted against the inverse temperature. This allowed the extraction of the activation energy $E_{A}$ as a metric for the temperature dependency of the currents [44]. Thereby, a lower activation energy implies a higher heat tolerance. It turned out that, in the on-state of the cell, $E_{A}$ was 37meV (at $R_{\mathrm{on},p}$) and 96meV in the off-state (at $R_{\mathrm{off}}$). In comparison to other low-dimensional nanomaterials such as platinum diselenide (50meV–200meV) [45,46], molybdenum disulfide (≈570meV) [47], and hexagonal boron nitride (≈450meV) [47], the $E_{A}$ for our CNT-PUFs was rather small. Next, the activation energy was extracted from 81 non-conductive cells. These currents originated from leakage between the gate and source/drain electrodes, as well as from measurement noise. There, a low average $E_{A}$ of 12meV was obtained. This implies an even lower temperature dependence compared to conductive cells. Consequently, our CNT-PUF cells featured an excellent heat tolerance characterised by activation energies lower than approximately 40meV for both binary states, conductive ($R_{\mathrm{on},p}$→ logical 1) and non-conductive ($R_{\mathrm{non}-\mathrm{conductive}}$ → logical 0).

## 6. Stable Key Extraction from CNT-PUFs

As shown in the previous section, extracting a CNT-PUF response from raw measurements without subsequent post-processing yielded already nearly optimal results in terms of robustness, uniqueness, and unpredictability, even at temperatures up to $120\;{}^{\circ }C$. Despite these promising results, additional post-processing is required to use the CNT-PUF responses in cryptographic applications. This is due to the so-called avalanche effect, which is a requisite for cryptographic encryption algorithms and ensures that even a slight change in a key leads to vastly distinct ciphertexts or plaintexts. This, in turn, requires the cryptographic key computed from the PUF to be completely stable [48]. The minor errors observed during quantisation (as discussed in Section 5.2.4) require a lightweight post-processing procedure. To rectify these bit errors in the raw PUF response, we used a helper data scheme, which selects stable bits from the raw PUF response. The proposed method was demonstrated to serve as secure key storage, but can easily be adapted to a variety of different use cases.

### 6.1. Enrolment

The enrolment phase must be executed in a secure environment. This ensures that no malicious adversary may attempt to eavesdrop or manipulate the PUF readout while executing this phase. During enrolment, the raw PUF measurements $M_{p}^{set}$ are acquired, consisting of multiple $I_{D,\max,p}$ measurements of all PUF cells of a PUF *p*. These measurements are used to generate helper data represented as a list of indices to stable PUF cells. This list serves as a challenge $C_{p}^{sel}$. The enrolment is described in the procedure denoted in Algorithm 1.
**Algorithm 1** Retrieving a challenge as a list of cell indices of stable cells of a CNT-PUF *p*. 1:**procedure**Enrolment($M_{p}^{set},I_{th},L_{p}^{cid},n_{bits}$) 2:    **return**$:\;C_{p}^{sel}$   3:    $M_{p}^{min}\leftarrow min\left(M_{p}^{set}\right)$ 4:    $M_{p}^{max}\leftarrow max\left(M_{p}^{set}\right)$ 5:    $L_{th}^{min}\leftarrow \emptyset$ 6:    **for** $c_{id}\in L_{p}^{cid}$ **do** 7:        $r_{p}^{i}\leftarrow Quantise\left(M_{p}^{min}\left[c_{id}\right],I_{th}\right)$ 8:        $r_{p}^{j}\leftarrow Quantise\left(M_{p}^{max}\left[c_{id}\right],I_{th}\right)$ 9:        **if** $r_{p}^{i}==r_{p}^{j}$ **then**10:           $d_{min}\leftarrow min([abs\left(I_{th}-M^{min(i)}\left[c_{id}\right]\right),$11:           $\;\;\;\;\;\;\;\;\;\;\;\;\;\;\;\;\;\;\;\;\;\;abs(I_{th}-M^{max(i)}\left[c_{id}\right])])$12:           $L_{th}^{min}\to append\left(\left(c_{id},r_{p}^{i},d_{min}\right)\right)$13:        **end if**14:    **end for**15:    16:    $L_{d_{min}}^{ordered}\leftarrow order\left(L_{th}^{min},d_{min}\right)$17:    $L_{0},L_{1}\leftarrow split\left(L_{d_{min}}^{ordered},r_{p}\right)$18:    $C_{p}^{sel}\leftarrow SelectRandomPUFCells\left(L_{0},L_{1},n_{bits}\right)$19:**end procedure**

This algorithm utilises a list of measurements $M_{p}^{set}:=\left\{M_{p}^{0},\ldots M_{p}^{n-1}\right\}$ for each of the *n* PUF cells and a threshold $I_{th}$. It additionally requires a list of cell indices $L_{p}^{cid}:=\left\{c_{id}^{0},\ldots ,c_{id}^{n-1}\right\}$ of all cells contained in $M_{p}^{set}$, as well as $n_{bits}\in \;\left\{0,\ldots ,n\right\}$ representing the desired length of the generated key. In the first step, the measurements with the lowest ($min(M_{p}^{i})$) and the highest ($max(M_{p}^{i})$) source–drain current of each cell *i* are extracted and stored in separate lists $M_{p}^{min}:=\left\{min\left(M_{p}^{0}\right),\ldots ,min\left(M_{p}^{n-1}\right)\right\}$ and $M_{p}^{max}:=\left\{max\left(M_{p}^{0}\right),\ldots ,max\left(M_{p}^{n-1}\right)\right\}$, respectively. Each PUF consists of n=144 cells in this work. Subsequently, a quantisation of each cell’s maximum and minimum values identified by $c_{id}$ is performed using the quantisation algorithm described in Section 4.3. The resulting binary values are stored in the variables $r_{p}^{i}$ and $r_{p}^{j}$. If both values are identical, this indicates that the maximum and minimum values of all repeated measurements of a cell are located on the same side of $I_{th}$, meaning that all intermediary measurements would be on the same side as well. Therefore, the cell can be reliably classified. The generated set of cell indices is stored in a list and ordered according to the distance $d_{min}$ from the threshold $I_{th}$, yielding a list $L_{d_{min}}^{ordered}$. This list is further split into two new lists $L_{0}$ and $L_{1}$, where $L_{0}$ contains the indices of cells assigned to logical 0, ordered by distance $d_{min}$, and $L_{1}$ contains the corresponding cells assigned to logical 1.

Finally, we merged the CNT-PUF cells in Algorithm 2, yielding random responses of 0 s and 1 s, which assemble a PUF response.
**Algorithm 2** Selection of random PUF cells using a Random Number Generator (RNG). 1:**procedure**SelectRandomPUFCells($L_{0}$, $L_{1}$, $n_{bits}$) 2:    **return**$:\;C_{p}^{sel}$   3:    **for** $i\in \left[0,1,\ldots ,n_{bits}-1\right]\wedge i<|L_{0}\left|\wedge i<\right|L_{1}|$ **do** 4:        $\delta \in_{R}\left\{0,1\right\}$ 5:        **if** δ==0 **then** 6:           $\left(c_{id},r_{p}^{i},d_{min}\right)\leftarrow pop\left(L_{0}\right)$ 7:        **else** 8:           $\left(c_{id},r_{p}^{i},d_{min}\right)\leftarrow pop\left(L_{1}\right)$ 9:        **end if**10:        $C_{p}^{sel}\to append\left(c_{id}\right)$11:    **end for**12:**end procedure**

In this procedure, we assumed a Random Number Generator (RNG) that produces unpredictable and uniformly distributed random numbers $\delta \in_{R}\left\{0,1\right\}$. The algorithm always selects the cell with the most-significant distance to $I_{th}$ either from $L_{0}$ if δ=0 or from $L_{1}$, otherwise, until $n_{bits}$ are generated, or one of the lists $L_{0}$ or $L_{1}$ is exhausted. The resulting list of cell indices forms the challenge $C_{p}^{sel}$ of the CNT-PUF. The cells with the greatest distance from $I_{th}$ are selected first because these cells indicate the highest stability and, thus, the lowest probability that some of the future responses will be located on the opposite side of $I_{th}$.

The enrolment process must occur in a secure environment, typically after manufacturing. Furthermore, $C_{p}^{sel}$ is transferred via a secure channel to the entity that will store the challenge for later use in requesting the PUF response during the reconstruction phase. As is the case for any other helper data scheme, the integrity of the challenge $C_{p}^{sel}$ must be preserved. One method could be using message authentication codes to prevent unauthorised modifications of the helper data. Furthermore, we were able to generate fully stable responses based on the available dataset, making the response suitable for cryptographic applications. Because not only the distribution of conducting and non-conducting cells is random, but also the distance from the threshold $I_{th}$ is unpredictable as well, the cell selection from $L_{0}$ and $L_{1}$ leaks no vulnerable information. Note that this algorithm is only executed once, typically after manufacturing. Therefore, the cells can be measured and ordered utilising high-resolution analogue–digital converters. Subsequently, the generated helper data are converted into a challenge. The reconstruction process, executed during runtime, is very lightweight, involving only comparison measurements with a fixed threshold, which can be implemented using a significantly less-complex circuit. A detailed explanation of this process is provided in the subsequent section.

### 6.2. Reconstruction

During the reconstruction phase, a stable key is reproduced from a CNT-PUF *p* based on the list of stable cells $C_{p}^{sel}$, serving as a challenge generated during the enrolment. The algorithm reconstructing the key from a PUF is outlined in Algorithm 3.

The CNT-PUF response is generated by iteratively measuring all cells corresponding to the cell indices $c_{id}\in C_{p}^{sel}$. Subsequently, a quantisation operation is executed, as outlined in Section 4.3, resulting in a logical 0 or logical 1 for each selected cell. There is no necessity to gather multiple measurements of the cells, as a simple comparison of each cell measurement with $I_{th}$ is sufficient to assign a binary value to that cell. These binary values are accumulated in a list $R_{p}$, which constitutes the response of the PUF *p*, which can further be used, for example, as a key in a cryptographic application.
**Algorithm 3** Algorithm describing the reconstruction phase of a CNT-PUF *p*.1:**procedure**Reconstruction($C_{p}^{sel},I_{th}$)2:    **return**$:\;R_{p}$  3:    $R_{p}\leftarrow \emptyset$4:    **for** $c_{id}\in C_{p}^{sel}$ **do**5:        $m_{i}\leftarrow Measure\left(c_{id}\right)$6:        $r_{i}\leftarrow Quantise\left(m_{i},I_{th}\right)$7:        $R_{p}\to append\left(r_{i}\right)$8:    **end for**9:**end procedure**

## 7. Conclusions

In this work, we presented a design for CNT-PUFs and revealed their properties for use under different temperatures. The reason for those excellent properties lies in the statistical distribution of the maximum current for the cells of the CNT-PUF, which form two clearly separated clusters. Furthermore, the size of these clusters, i.e., the number of cells with the respective maximum current, is directly adjustable by the fabrication process and the device layout. In conclusion, our raw CNT-PUF responses yielded nearly optimal results for all evaluated PUF metrics. This was demonstrated by a high level of uniqueness, as evidenced by an average inter-device Hamming distance of 0.48. A nearly uniform distribution of the PUF responses could be achieved by a ratio of 55.29% conducting and 44.71% non-conducting PUF cells. Furthermore, the PUF responses were found to be highly unpredictable through an evaluation utilising the well-known NIST SP 800-22 test suite.

In repeated measurements at $23\;{}^{\circ }C$ and in the temperature range between $40\;{}^{\circ }C$ and $120\;{}^{\circ }C$, the CNT-PUF responses were very robust, exhibiting an error rate of at most 2%. We attributed this excellent heat tolerance to the comparatively low extracted activation energies of less than 40meV for the relevant binary states. Thus, our CNT-PUF architecture is able to generate robust security primitives in environments significantly exceeding the temperature limits of $70\;{}^{\circ }C$ for commercial semiconductor devices and $85\;{}^{\circ }C$ for industrial semiconductor devices. Furthermore, compared to other works in the relevant literature, we presented a CNT-based PUF design operating on passivated structures, further improving the robustness against environmental influences such as changes in the atmosphere around the device. The passivation, combined with the atomic sizes of CNTs and the low CNT density, makes them impervious to various attacks, like probing by optical microscopes. To safeguard against attacks based on the use of electron microscopes, additional metal layers could be added on top of the CNT-PUF.

Finally, small remaining instabilities could easily be solved by a very lightweight post-processing scheme, in contrast to solutions in the existing literature that rely on computationally heavy error-correcting codes and fuzzy extractors. Despite the simplicity of the presented post-processing scheme, the algorithm allows stability errors to be fully corrected, resulting in a perfectly stable response with negligible overhead in terms of computing resources.

The presented measurements were obtained using a highly sensitive parameter analyser. However, for practical use cases, integrated electronic components, such as amplifier circuits, analogue–digital conversion, and multiplexers are needed in order to allow efficient quantisation for large bit strings. For this reason, we reserve the demonstrated CNT-PUF architecture for heterogeneous system integration technologies such as chiplet or monolithic System-on-a-Chip (SoC) technologies connected to front-end electronics inherently offering these components. Furthermore, such a configuration also enables the nanomaterials to reveal the variety of their properties as they can take over multiple tasks for forthcoming cost-efficient hybrid electronic systems.

## Figures and Tables

**Table 1:** Average *p*-values and amount of passed tests for the NIST SP 800-22 test suite [24] applied on each individual CNT-PUF response of channel width 1000 nm, with a sequence length of n=144.

Statistical Test	Average *p*-Value	Passed/Total	Input Length Recommendation Test Parameters
Frequency (Monobit)	0.28	25/30	n≥ 100 bits
Frequency Test within a Block	0.32	28/30	n≥ 100 bits *M* = 20 bits (block length)
Runs Test	0.43	30/30	n≥ 100 bits
Test for the Longest Run of Ones in a Block	0.41	27/30	n ≥ 128 bits *m* = 8 bits (block length)
Binary Matrix Rank Test	/	/	n ≥ **38,912 bits**
Discrete Fourier Transform (Spectral) Test	/	/	n ≥ **1000 bits**
Non-overlapping Template Matching Test	0.86	30/30	*m* = 9 bits (template size)
Overlapping Template Matching Test	/	/	n ≥ $10^{6}$ **bits**
Maurer’s “Universal Statistical” Test	/	/	n ≥ **387,840 bits**
Linear Complexity Test	/	/	n ≥ $10^{6}$ **bits**
Serial Test	0.36	30/30	$m<[\log_{2}n]-2$ *m* = 4 bits (block length)
Approximate Entropy Test	0.33	27/30	$m<[\log_{2}n]-5$ *m* = 2 bits (block length)
Cumulative Sums (Cusum) Test	0.30	26/30	n≥ 100 bits
Random Excursions Test	/	/	n ≥ $10^{6}$ **bits**
Random Excursions Variant Test	/	/	n ≥ $10^{6}$ **bits**

## Data Availability

The experimental data produced for the purposes of this work are available on reasonable request from the corresponding authors.

## Supplementary Materials

The following supporting information can be downloaded at: https://www.mdpi.com/article/10.3390/nano13222930/s1, Figure S1: Absorbance of CNT dispersion and visualization of the sub-band transition energy branches for semiconducting (S22 + S33) and metallic (M11) CNTs limited to the diameter specifications provided by NanoIntegris Technologies Inc. [40]. Solvent peaks from toluol are subtracted; Figure S2: Dependence of CNT line density on printing velocity for a flow rate of 0.4 μL/min; Figure S3: Histogram of the drain current for CNT-FET arrays with a channel width of 2000 nm before and after passivation. The insets show the converted binary distribution of conductive and non-conductive cells. Reference [40] is also cited in the Supplementary Materials file.

## Abbreviations

The following abbreviations are used in this manuscript:

AFM	Atomic Force Microscopy
ALD	Atomic Layer Deposition
ASIC	Application-Specific Integrated Circuit
CMOS	Complementary Metal–Oxide–Semiconductor
CNT	Carbon NanoTube
CNT-FET	Carbon NanoTube Field-Effect Transistor
CNT-PUF	Carbon NanoTube-based Physical Unclonable Function
CVD	Chemical Vapour Deposition
EBL	Electron-Beam Lithography
FET	Field-Effect Transistor
IoT	Internet of Things
NIST	National Institute of Standards and Technology
PUF	Physical Unclonable Function
RNG	Random Number Generator
SoC	System on a Chip
