# Supplementary material for: CNT-PUFs: Highly Robust and Heat-Tolerant Carbon-Nanotube-Based Physical Unclonable Functions
Source: Nanomaterials (Basel). 2023 Nov 11;13(22):2930. doi: 10.3390/nano13222930 (PMC10674552; doi:10.3390/nano13222930)
Supplement: Supplementary file 1 [file nanomaterials-13-02930-s001.zip › main.pdf]

Supplementary Materials: CNT-PUFs: Highly Robust and Heat-Tolerant Carbon-Nanotube-Based Physical Unclonable Functions

Florian Frank, Simon Böttger, Nico Mexis, Nikolaos Athanasios Anagnostopoulos, Ali Mohamed, Martin Hartmann, Harald Kuhn, Christian Helke, Tolga Arul, Stefan Katzenbeisser and Sascha Hermann

Supplementary Materials

This manuscript contains supplementary information not presented in the primary manuscript, providing additional measurement data and corresponding descriptions.

1. Wafer-level Fabrication and Characterization

This section contains additional information related to Section 4.1 of the main document, such as detailed information on the CNT raw material, the CNT integration process, and the influence of CNT-FET passivation on the electrical CNT-PUF.

1.1. CNT Raw Material Dispersion and CNT Integration

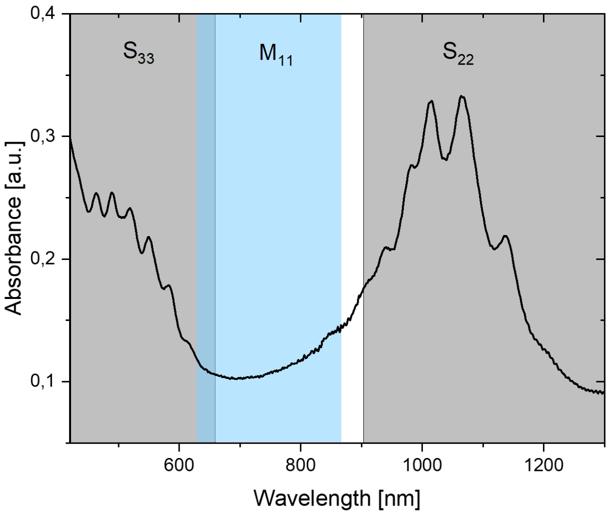

Figure S1. Absorbance of CNT dispersion and visualization of the sub-band transition energy branches for semiconducting (S22 + S33) and metallic (M11) CNTs limited to the diameter specifications provided by NanoIntegris Technologies Inc. [1]. Solvent peaks from toluol are subtracted.

Figure S1 shows the absorbance spectra of the CNT dispersion with transition energies limited to the diameter specifications [1] provided by NanoIntegris Technologies Inc. (located in Boisbriand, Quebec, Canada). The appearance of peaks solely related to semiconducting inter-band transitions proves the high enrichment grade of the CNTs. For further reading, we refer to the online-available datasheet of the used commercial CNT dispersions [1].

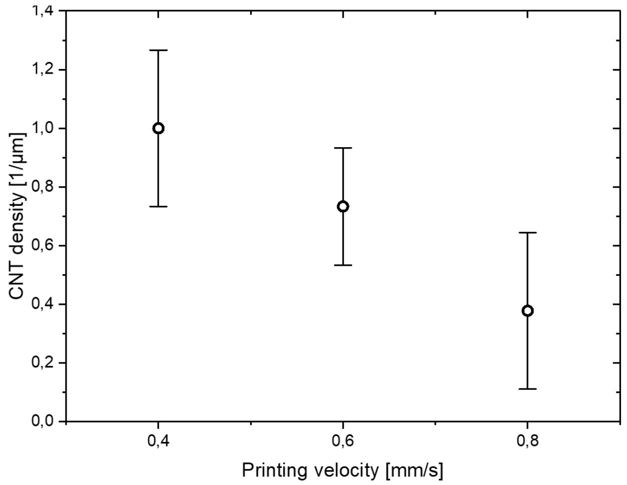

Figure S2. Dependence of CNT line density on printing velocity for a flow rate of 0.4 µL/ min.

The CNT-integration process is comparable to a zone-casting process. Therefore, a syringe pump is connected to a cannula to supply a continuous flow of toluene-based dispersion while the substrates laterally move at a distinct velocity. This creates printing tracks featuring stochastically aligned CNTs perfectly suited for CNT-PUFs. For this work, the CNT concentration was adjusted to ≈15 µg/mL.

1.2. Impact of Passivation on the Behavior of CNT-PUFs

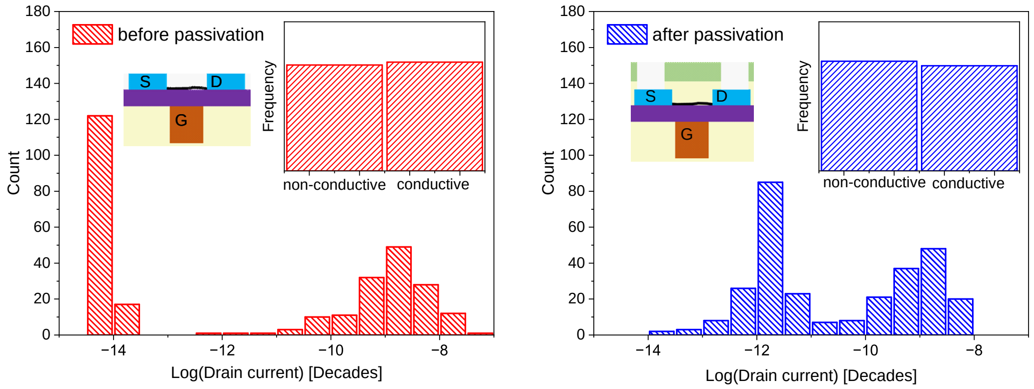

Figure S3. Histogram of the drain current for CNT-FET arrays with a channel width of 2.000 nm before and after passivation. The insets show the converted binary distribution of conductive and non-conductive cells.

Figure S3 shows the distribution of the maximal current $I_{D,\mathrm{max,p}}$ before and after passivation. It can be seen that the samples before passivation exhibit an even higher separation between conductive and non-conductive devices. This can probably be attributed to changed material properties induced by the passivation layer grown by chemical-vapor deposition, such as increased conductivity of the surrounding dielectric. Additionally, the integration time of the measurement, as well as the induced charge traps are assumed to have decisive influences. However, we were able to achieve highly distinguishable classes of CNT-FETs, ensuring robustness, which actually supports the findings included in the main document of our work, where an average intra-device Hamming distance of 0.06% was observed when applying a threshold of $I_{th} = 20\,\mathrm{pA}$ under normal conditions.

References

1. Nanointegris Technologies Inc. IsoSol-S100® Polymer-Wrapped Nanotubes Technical Data Sheet. Available online: https://nanointegris.com/our-products/isosol-s100-polymer-wrapped-nanotubes/ (accessed on 26 October 2023).

Disclaimer/Publisher's Note: The statements, opinions and data contained in all publications are solely those of the individual author(s) and contributor(s) and not of MDPI and/or the editor(s). MDPI and/or the editor(s) disclaim responsibility for any injury to people or property resulting from any ideas, methods, instructions or products referred to in the content.
